# Polymorphism in a secondary phosphine

**DOI:** 10.1107/S2053229625000555

**Published:** 2025-01-30

**Authors:** Mo Liu, Keith Izod, Paul G. Waddell

**Affiliations:** a School of Natural and Environmental Sciences, Bedson Building, Newcastle University, Newcastle upon Tyne, NE1 7RU, United Kingdom; J-PARC Center, Japan Atomic Energy Agency, Japan

**Keywords:** crystal structure, secondary phosphine, polymorphism, crystal packing, Me⋯π inter­actions, steric pressure

## Abstract

Two crystal structures of bis­(2,3,5,6-tetra­methyl­phen­yl)phosphine, C_20_H_27_P, are reported constituting the first recorded case of polymorphism in a secondary phosphine (*R*_2_PH). The two structures differ in their conformation and, as a result, the steric hindrance experienced at the phospho­rus centre is observed to be dependent on the packing environment. Each polymorph exhibits a distinct supra­molecular structure; in polymorph **I** the mol­ecules are arranged in columns in two directions, whereas polymorph **II** forms layers. There is a distinct lack of significant inter­molecular inter­actions in either form, with the exception of some weak Me⋯π inter­actions observed in polymorph **II**. These inter­actions are likely the cause of the variation in the C—P—C angles observed between the two structures.

## Abstract 2

Bis(2,3,5,6-tetra­methyl­phen­yl)phosphine is the first secondary phosphine known to exhibit polymorphism and is observed to form two different crystal structures depending on the solvent of crystallization. As structures of these reactive com­pounds are somewhat rare in the literature, this study expands the sum of structural knowledge of secondary phosphines, as well as revealing aspects of their supra­molecular chemistry.

## Introduction

Phosphines have become ubiquitous ligands for transition-metal centres due to the ease with which their electronic and steric properties may be tailored and to the many and varied applications of transition-metal phosphine com­plexes in catal­y­sis. Secondary phosphines *R*_2_PH have the added advantage that the P—H proton may readily be removed to furnish anionic phosphanide *R*_2_P^−^ ligands (Izod, 2000 ▸). We have a long-standing inter­est in the application of such phos­phanide ligands for the support of novel low-oxidation-state main group species; for example, the recently isolated fully phosphanyl-substituted ditetrelenes {(Mes)_2_P}_2_*E*=*E*{P(Mes)_2_}_2_ (*E* = Si or Ge; Mes = 2,4,6-Me_3_C_6_H_2_) (Izod *et al.*, 2017*a* ▸, 2022 ▸).

In the course of this work, we have striven to explore the impact of the steric profile and substitution pattern of the aromatic rings in di­aryl­phosphanide ligands on the structures and stabilities of both low-oxidation-state main group com­pounds and their alkali metal precursors *R*_2_P*M* (*M* = Li, Na or K) (Izod *et al.*, 2017*b* ▸). While 2,6-disubstituted and 2,4,6-tri­sub­stituted aromatic rings are common phosphine substituents, alternative substitution patterns, such as in the 2,3,5,6-tetra­methyl­phenyl substituent described here, are rare.

Due to their reactivity, the structure determination of sec­ondary phosphines using single-crystal X-ray crystallography can be challenging, with the first such structure being reported in 1987 (Bartlett *et al.*, 1987 ▸). As testament to this, at time of writing there are only 95 organic acyclic secondary phosphine structures in the Cambridge Structural Database (CSD, Ver­sion 5.45, update 2, June 2024; Groom *et al.*, 2016 ▸) and only one polymorph is reported for any one secondary phosphine com­pound, including cyclic phosphines and organometallic com­plexes.

**Figure 1:**
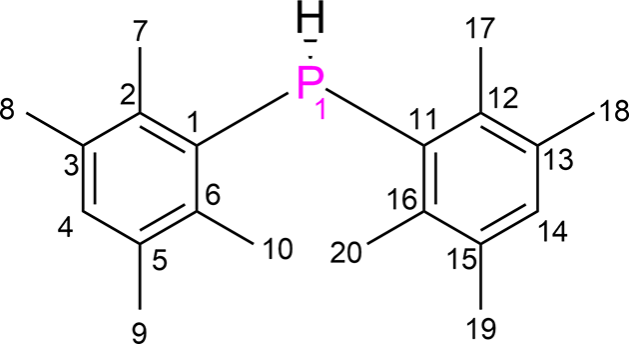
Bis(2,3,5,6-tetra­methyl­phen­yl)phosphine with the numbering scheme used in this article.

In this work, we present the first known instance of polymorphism in a secondary phosphine. Bis(2,3,5,6-tetra­methyl­phen­yl)phosphine (Fig. 1 ▸) crystallizes in two distinct forms: polymorph **I**, grown from tetra­hydro­furan, which crystallizes in the monoclinic space group *P*2/*n*, and polymorph **II**, grown from fluoro­benzene, which crystallizes in the monoclinic space group *P*2_1_/*c*. As the first case of its kind, the structural analysis here should provide unique insights into the supra­molecular chemistry of secondary phosphines.

## Experimental

### Preparation of bis­(2,3,5,6-tetra­methyl­phen­yl)phosphine

All manipulations were performed under an inert atmosphere (argon gas) using standard Schlenk techniques unless otherwise stated. To a cold (−78 °C) solution of PCl_3_ (2.9 ml, 23 mmol) in diethyl ether (50 ml) was added (2,3,5,6-Me_4_C_6_H)MgBr (42 mmol) dissolved in tetra­hydro­furan (THF, 200 ml). This mixture was allowed to warm to room tem­per­a­ture and was stirred for 12 h. To this solution was carefully added an excess of solid LiAlH_4_ (1.0 g, 26.3 mmol) and the resulting mixture was stirred at room tem­per­a­ture for 2 h. Degassed water (50 ml) was carefully added and the organic phase was extracted into THF (3 × 30 ml). The combined organic extracts were dried over activated 4 Å mol­ecular sieves, the solution was filtered and solvent was removed *in vacuo* from the filtrate to give bis­(2,3,5,6-tetra­methyl­phen­yl)phosphine as a colourless solid in 65% yield. Crystals suitable for single-crystal X-ray diffraction were grown from cold (3 °C) THF (polymorph **I**) or from cold (−30 °C) fluoro­benzene (polymorph **II**).

### Refinement

Crystal data, data collection and structure refinement details are summarized in Table 1 ▸. H atoms bound to C atoms were positioned with idealized geometry. The displacement parameters of these H atoms were constrained using a riding model, with *U*_iso_(H) values set to be an appropriate multiple of the *U*_eq_ value of the parent atom.

The H atoms bound to phospho­rus were located using peaks in the Fourier difference map. In both structures, the most prominent residual peaks about phospho­rus after all other atoms were modelled were selected. In the case of polymorph **I**, the occupancy of this H atom was constrained to be 0.5 as it is disordered across a special position. For polymorph **II**, peaks corresponding to two proton positions with similar geometry were observed and hence the phosphine H atom was split across two positions with the occupancies refined to be approximately 63 and 37%. The displacement parameters of the phosphine H atoms in both structures were constrained using a riding model, with *U*_iso_(H) values set to be 1.2*U*_eq_ relative to the parent atom.

It is likely that the unrestrained P—H distances are shorter than the true bond lengths, but the direction of the bond vectors are likely to be accurate. Though some residual density remains, most prominently in the structure of polymorph **II**, there are no peaks greater than 0.5 e Å^−3^ and they do not appear to be in positions that could correspond to atoms; the largest peak is altogether too close to the P atom (<1 Å) and/or in a position that would make little sense in terms of mol­ecular geometry. It is possible that these residual peaks are the result of series termination errors (Fourier ripples).

## Results and discussion

The two structures of bis­(2,3,5,6-tetra­methyl­phen­yl)phosphine crystallize in different monoclinic space groups. Polymorph **I** crystallizes in the space group *P*2/*n*, with an asymmetric unit com­prising half of the mol­ecule (*Z*′ = 0.5). Here the P atom is located on the twofold rotation axis in the structure and the full mol­ecule is generated through this symmetry operation. Polymorph **II** crystallizes in the space group *P*2_1_/*c*, with one whole mol­ecule in the asymmetric unit. In both structures, the proton on the P atom is disordered over two positions, as has been observed previously in similar bis­(ar­yl) secondary phosphine structures (Izod *et al.*, 2017*b* ▸; Clegg, 2017 ▸). Details of the refinements for both structures are presented in Table 1 ▸.

**Figure 2:**
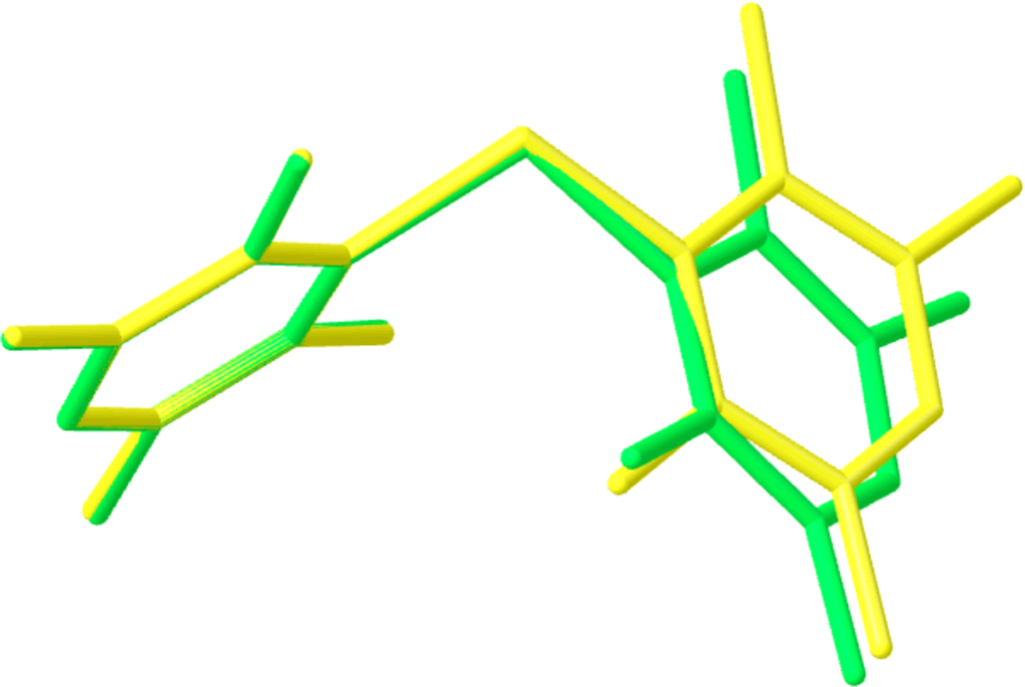
Overlay diagram of polymorphs **I** (yellow) and **II** (green).

Though the bond distances do not differ significantly, the conformations of the mol­ecules in the two polymorphs are somewhat different, as demonstrated by overlaying them (Fig. 2 ▸). The conformational variation can be attributed to differences in the geometry about the P atom and the angles between the planes of the aryl rings (Table 2 ▸). As polymorph **I** exhibits a wider C—P—C angle than polymorph **II**, this would suggest that it experiences a greater degree of steric hindrance at the phospho­rus centre (Rivard *et al.*, 2007 ▸).

The degree of steric pressure on the P atom in bis­(ar­yl)phosphines can also be assessed by the sum of the angles about phospho­rus, Σ°P (Boeré & Zhang, 2005 ▸). The values measured exceed 300°, with polymorph **I** exhibiting a Σ°P of 318 (2)° and the same sum being 333.2 (2)° for polymorph **II** (measured for the H atom of highest occupancy). This would seem to contradict the inter­pretation of the C—P—C bond angles as, according to the Σ°P, the P atom in polymorph **II** is under greater steric pressure in spite of its narrower C—P—C angle. Regardless of the trend in these measurements, that there should be such variation within the same mol­ecule demonstrates the effect that polymorphism can potentially have on these com­pounds. These conformational perturbations are likely the result of the different packing environments and inter­molecular inter­actions in the two solid-state structures.

As is common in the structures of secondary bis­(ar­yl)phosphines, there are no significant contacts involving the H atom on the phospho­rus in either structure (Izod *et al.*, 2017*b* ▸; Bartlett *et al.*, 1987 ▸; Clegg, 2017 ▸; Rivard *et al.*, 2007 ▸; Fleming *et al.*, 2013 ▸; Ritch *et al.*, 2014 ▸). The lack of structure-directing inter­actions involving this atom may well be the root of the disorder of the P—H proton manifest in both polymorphs.

**Figure 3:**
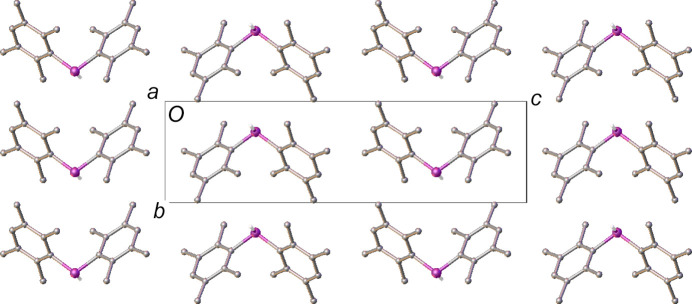
The structure of polymorph **I**, viewed along the [100] direction. Only one orientation of the H atoms bound to phospho­rus is shown and the rest have been omitted for clarity.

The packing in both structures seems to prioritize the minimization of steric inter­actions as opposed to forming strong structure-directing inter­molecular bonds. As such, the packing is best described in terms of the alignment of the aryl rings. The mol­ecules in polymorph **I** stack forming continuous columns along both the crystallographic [100] direction, with P⋯P distances of *ca* 6.55 Å (Fig. 3 ▸), and along the [010] direction, with an equivalent distance of *ca* 5.99 Å (the lengths of the respective axes). The rings exhibit similar angles to their respective directions, *ca* 57° in [100] and *ca* 52° in [010].

**Figure 4:**
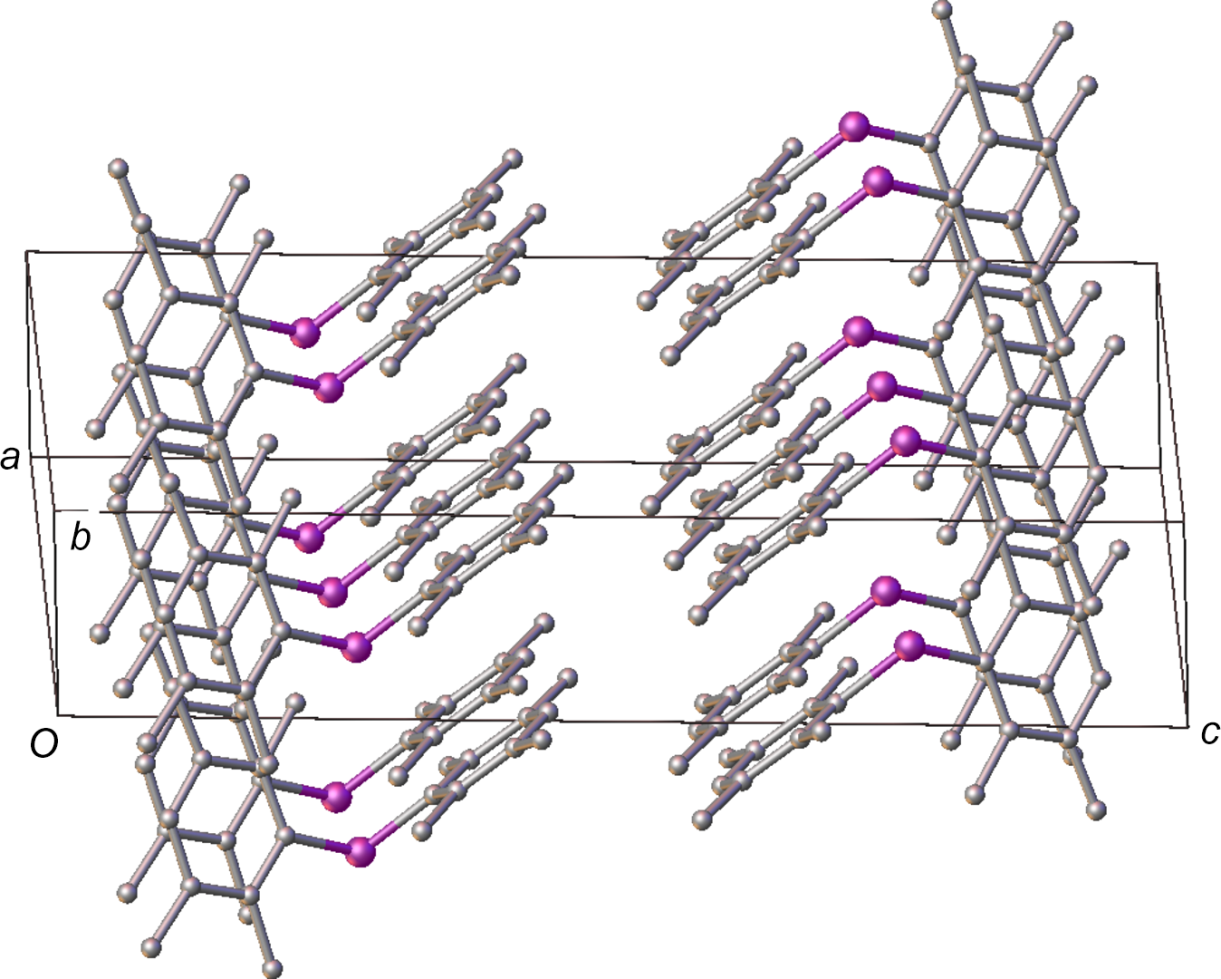
The structure of polymorph **I**, viewed approximately along the [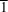
10] direction, showing the direct alignment of the methyl groups hindering close approach of the mol­ecules in this direction. H atoms have been omitted for clarity.

The distance between the mol­ecules along the columns appears to preclude direct π–π inter­actions (Avashti *et al.*, 2014 ▸; Brunner *et al.*, 2014 ▸). In fact, there do not appear to be any salient inter­molecular inter­actions observed in polymorph **I**. This suggests that the mol­ecules are arranged in such a way as to minimize repulsive contacts rather than form attractive inter­actions. As a result, all the duryl rings in this structure are orientated coplanar to either the [110] or [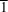
10] directions. The orientation of the rings in these directions, with methyl groups directed towards each other in the same plane, further hinders the close approach of the mol­ecules in the structure (Fig. 4 ▸).

The packing in the structure of polymorph **II** is markedly different to that in polymorph **I** and much of this can be attributed to the fact that the two aryl rings are crystallographically independent in polymorph **II**. The mol­ecules align along the [010] direction, but the angles of the rings to this direction are shallower than in polymorph **I**; *ca* 28° for one and 0° for the other, where, once again, the methyl groups prevent the close approach of the π systems. Though the arrangement of the rings in the (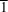
02) plane, propagating along [010], is reminiscent of a similar arrangement in polymorph **I**, also in the [010] direction, in polymorph **II** the spacing between the mol­ecules in this direction is over 2 Å longer, with a P⋯P distance of *ca* 8.85 Å along [010], likely the result of the shallower P—C—P angle.

**Figure 5:**
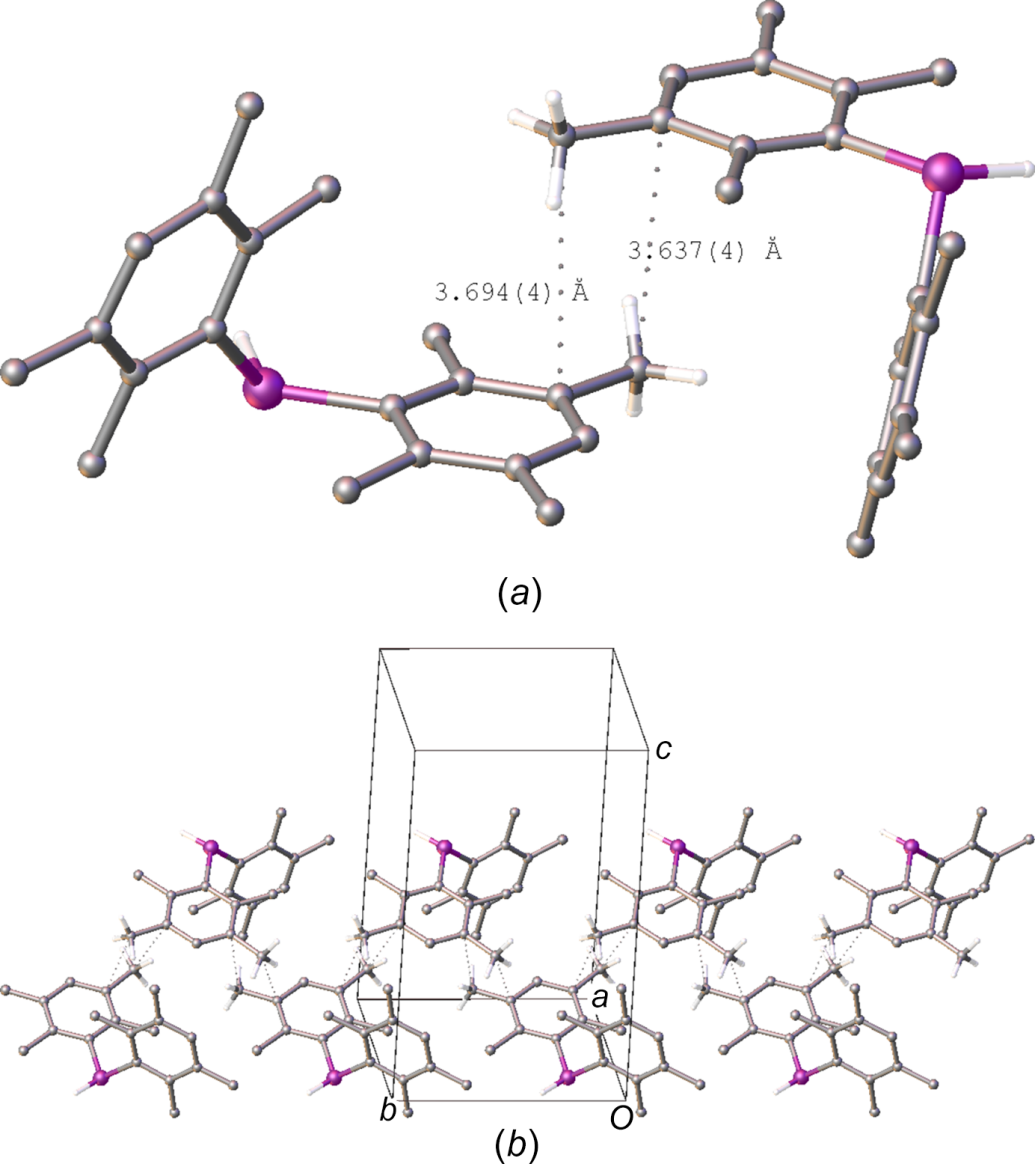
(*a*) A view of the ring motif formed in polymorph **II** of Me⋯π inter­actions between two mol­ecules and (*b*) the continuous chain motif formed of these inter­actions in the [010] direction. Close contacts are depicted as dashed lines and H atoms, with the exception of those bound to phospho­rus and the methyl groups involved in inter­molecular inter­actions, have been omitted for clarity.

In contrast to the structure of polymorph **I**, there do appear to be some weak inter­molecular inter­actions in the structure of polymorph **II** in the form of C—H⋯C contacts between methyl groups and the aromatic rings. Two such contacts, with C⋯C distances < 3.7 Å, are observed (Table 3 ▸), which can be classified as weak Me⋯π inter­actions (Brunner *et al.*, 2014 ▸). The two contacts form a ring motif between two of the duryl rings and propagate along the [010] direction, forming a chain of inter­molecular inter­actions, with each mol­ecule related to the next by the symmetry of the 2_1_ screw axis (Fig. 5 ▸).

By way of com­parison, a similar relationship between the mol­ecules is observed in both the [100] and [010] directions in polymorph **I**; however, in this case, the C⋯C distances are at least 0.1 Å too long to be considered Me⋯π inter­actions. Given this, it is possible that the weak but nonetheless attractive inter­actions observed in polymorph **II** are the root of the shallower C—P—C angle observed in this structure com­pared to that of polymorph **I**.

**Figure 6:**
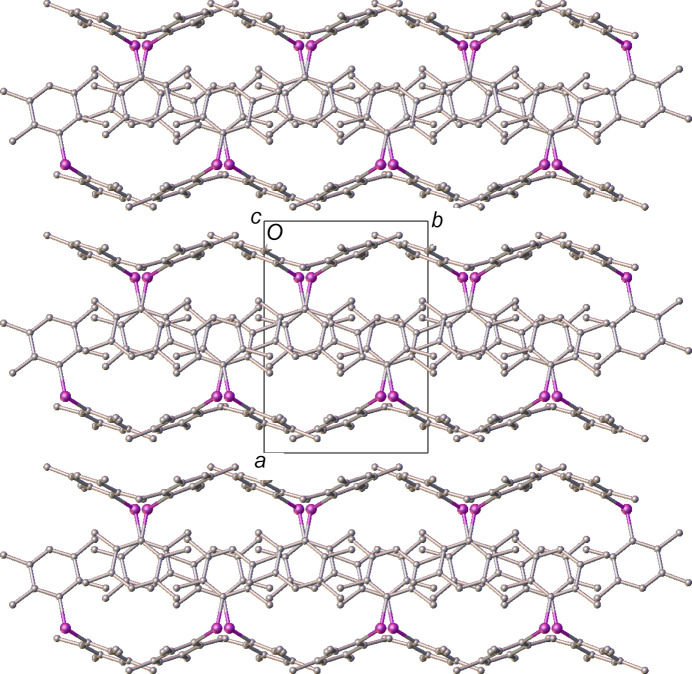
A view of the packing in polymorph **II**, showing the layers coplanar with the crystallographic (100) plane. H atoms have been omitted for clarity.

Although there are no discernible close contacts between the chains of mol­ecules in polymorph **II**, they appear to pack to form 2D layers coplanar to [100] (Fig. 6 ▸). Again, there do not appear to be any significant structure-directing inter­actions between these layers and the closest centroid–centroid distances between pairs of duryl rings across the layer boundary are *ca* 4.2 Å. As a result, it can be inferred that these rings are orientated simply to minimize steric inter­actions.<!?tpb=-20pt>

## Conclusion

Bis(2,3,5,6-tetra­methyl­phen­yl)phosphine is the first secondary phosphine known to exhibit polymorphism and is observed to form two different crystalline forms depending on the solvent of crystallization. As structures of these reactive com­pounds are somewhat rare in the literature, this study expands the sum of structural knowledge of secondary phosphines, as well as revealing aspects of their supra­molecular chemistry.

The mol­ecules in each crystal structure vary in terms of their conformation, with the degree of steric pressure on the P atom observed to vary depending on the packing environment. Though polymorph **I** crystallizes with columnar motifs in the [100] and [010] directions, and no significant structure-directing inter­molecular inter­actions, polymorph **II** forms a 2D layered structure with weak Me⋯π inter­actions, forming a chain motif along the [010] direction.

The study of polymorphism in a secondary phosphine raises some inter­esting points in terms of the solid-state structures of these mol­ecules. It should be noted that as the same mol­ecule exhibits drastically different values for Σ°P, a measure of the steric pressure on the P atom, that this is not an intrinsic mol­ecular property and can be affected by the packing environment. This demonstrates that caution should be exercised when drawing conclusions based on these values, especially in the context of solution-phase calculations.

## Supplementary Material

Crystal structure: contains datablock(s) kji190001_fa, kji190003_fa, global. DOI: 10.1107/S2053229625000555/oj3027sup1.cif

Structure factors: contains datablock(s) kji190001_fa. DOI: 10.1107/S2053229625000555/oj3027kji190001_fasup2.hkl

Structure factors: contains datablock(s) kji190003_fa. DOI: 10.1107/S2053229625000555/oj3027kji190003_fasup3.hkl

Supporting information file. DOI: 10.1107/S2053229625000555/oj3027kji190001_fasup4.cml

Molecular structures of the title polymprphs. DOI: 10.1107/S2053229625000555/oj3027sup5.pdf

CCDC references: 2405774, 2405773

## Figures and Tables

**Table 1:** Experimental details For both structures: C_20_H_27_P, *M*_r_ = 298.38. Experiments were carried out at 150 K with Cu *K*α radiation using a Rigaku Xcalibur Gemini ultra diffractometer with an Atlas detector. The absorption correction was analytical [*CrysAlis PRO* (Rigaku OD, 2015 ▸), based on expressions derived by Clark & Reid (1995 ▸)]. H atoms were treated by a mixture of independent and constrained refinement.

	Polymorph **I**	Polymorph **II**
Crystal data		
Crystal system, space group	Monoclinic, *P*2/*n*	Monoclinic, *P*2_1_/*c*
*a*, *b*, *c* (Å)	6.5476 (2), 5.9910 (2), 21.5676 (6)	12.8874 (6), 8.8455 (3), 15.4635 (7)
β (°)	96.020 (2)	104.108 (5)
*V* (Å^3^)	841.36 (4)	1709.61 (13)
*Z*	2	4
μ (mm^−1^)	1.35	1.33
Crystal size (mm)	0.24 × 0.08 × 0.06	0.36 × 0.14 × 0.05

Data collection		
*T*_min_, *T*_max_	0.696, 0.866	0.746, 0.939
No. of measured, independent and observed [*I* > 2σ(*I*)] reflections	11409, 1500, 1299	12709, 3021, 2424
*R*_int_	0.040	0.039
(sin θ/λ)_max_ (Å^−1^)	0.596	0.596

Refinement		
*R*[*F*^2^ > 2σ(*F*^2^)], *wR*(*F*^2^), *S*	0.039, 0.118, 1.06	0.047, 0.135, 1.06
No. of reflections	1500	3021
No. of parameters	103	214
No. of restraints	0	182
Δρ_max_, Δρ_min_ (e Å^−3^)	0.27, −0.24	0.43, −0.35

Computer programs: *CrysAlis PRO* (Rigaku OD, 2015 ▸), *SHELXT* (Sheldrick, 2015*a* ▸), *SHELXL* (Sheldrick, 2015*b* ▸) and *OLEX2* (Dolomanov *et al.*, 2009 ▸).

**Table 2:** Selected geometric parameters (Å, °) for polymorphs **I** and **II**

	Polymorph **I**	Polymorph **II**
P1—C1	1.8471 (17)	1.852 (2)
P1—C11		1.856 (2)
C1—P1—C1/11	108.78 (11)	105.74 (9)
C1—P1—H1	100 (2)	109.1 (2)
C1—P1—H1^i^/C11—P1—H1	99 (2)	118.4 (2)
Ar­yl–aryl twist angle	84.39 (10)	94.12 (9)

Symmetry code: (i) −*x* + 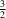, *y*, −*z* + 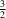.

**Table 3:** Inter­molecular Me⋯π inter­actions (Å, °) in the structure of polymorph

*D*—H⋯*A*	*D*⋯*A*	H⋯*A*	*D*—H⋯*A*
C18—H18*A*⋯C15^i^	3.694 (4)	2.83 (2)	150 (1)
C19^i^—H19*B*^i^⋯C15	3.637 (4)	2.88 (2)	136.6 (9)

Symmetry code: (i) −*x* + 1, −*y* + 2, −*z* + 1.
