# Supplementary material for: Polymorphism in a secondary phosphine
Source: Acta Crystallogr C Struct Chem. 2025 Jan 30;81(Pt 2):109–13. doi: 10.1107/S2053229625000555 (PMC11795654; doi:10.1107/S2053229625000555)
Supplement: Supplementary file 5 [file c-81-00109-sup5.pdf]

STRUCTURAL CHEMISTRY

Volume 81 (2025)

Supporting information for article:

Polymorphism in a secondary phosphine

Mo Liu, Keith Izod and Paul G. Waddell

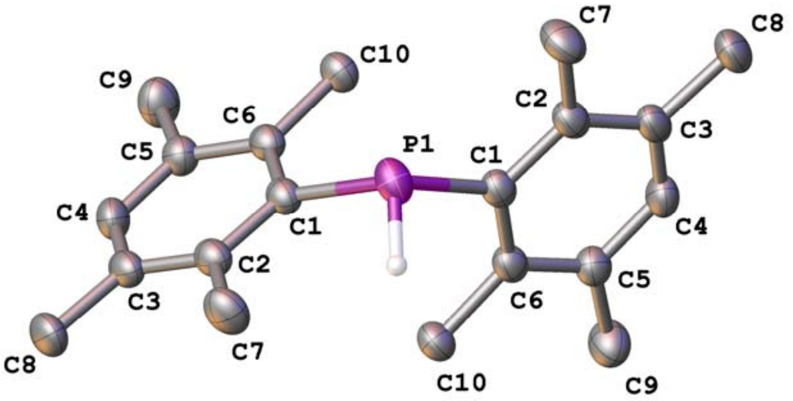

Fig. S1. The molecular structure of polymorph (**I**).

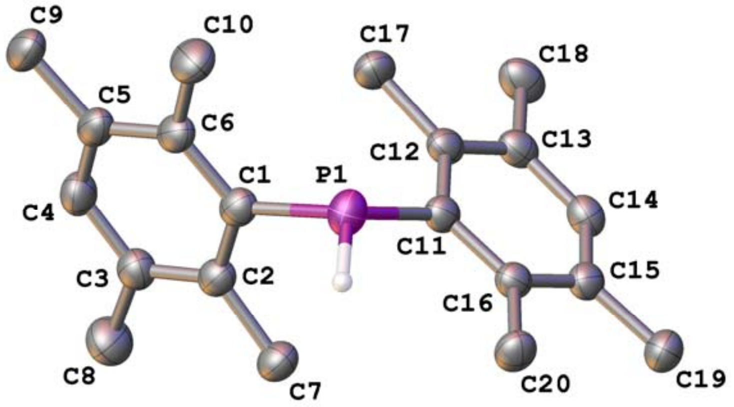

Fig. S2. The molecular structure of polymorph (**II**).
